# Extracellular vesicle long noncoding RNA as potential biomarkers of liver cancer

**DOI:** 10.1093/bfgp/elv058

**Published:** 2015-12-03

**Authors:** Swathi Mohankumar, Tushar Patel

**Keywords:** hepatocellular cancer, exosomes, extracellular vesicles, RNA genes, cancer diagnosis, digital PCR

## Abstract

Analysis of extracellular vesicles (EV) and their contents may be useful as disease biomarkers if they reflect the contents of cells of origin, differ between normal and diseased tissue and can be reliably detected. An increasing number of long noncoding RNA (lncRNA) are being reported to be aberrantly expressed in human cancers. These tumor-associated lncRNA may have potential as new biomarkers of disease. In this review, we highlight lncRNAs that are commonly associated with hepatocellular cancer, and summarize their potential biological roles and underlying molecular mechanisms. While lncRNA can be detected in the circulation, their low expression within circulating vesicles will require the use of highly sensitive detection technologies such as digital polymerase chain reaction or next-generation sequencing. While the integrity and functional role of tumor-specific lncRNAs within EV have yet to be established, their presence or enrichment within tumor cell-derived EV offers promise for their potential as disease biomarkers.

## Introduction

**Figure 1:**
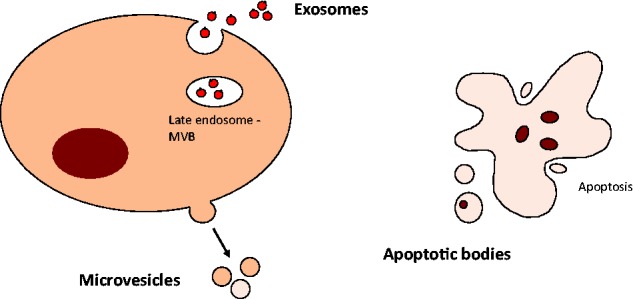
EVs. Several distinct types of vesicles are recognized. These include exosomes, microvesicles and apoptotic bodies. (A colour version of this figure is available online at: http://bfg.oxfordjournals.org)

Extracellular vesicles (EVs) are small membrane-bound vesicles that are released into the interstitial fluid from a wide variety of normal or diseased cells. The predominant types of EVs are exosomes, microvesicles and apoptotic bodies and these are distinguished on the basis of their size and biogenesis [1]. Exosomes have a diameter of 30–100 nm and are cell-derived vesicles that are thought to be released from intracellular multivesicular bodies. In contrast, microvesicles and microparticles range in size from 100 to 1000 nm, are released from the plasma membrane during cell stress via exocytic budding. Apoptotic bodies are larger, with a diameter of >1000 nm and are released from cells undergoing apoptosis (Figure 1). These vesicles contain a variety of proteins, lipids, RNA and DNA molecules [2]. Some of these may be selectively enriched within EVs during their formation. Recent studies have highlighted a key role of EVs in intercellular communication through the transfer of their contents such as RNA that can functionally modulate cellular activities in recipient cells [3]. The RNA content of EVs include messenger RNA (mRNA), microRNAs (miRNAs) and long noncoding RNAs (lncRNAs) [2]. Different types of EVs have been detected in many different body fluids. Consequently, they may potentially contribute to distant communication, an intriguing proposition that warrants systematic study.

EVs can be detected and isolated from body fluids. Analysis of EVs and their contents may have additional utility as disease biomarkers if they reflect the contents of cells of origin, differ between normal and diseased tissue and can be reliably detected. LncRNAs are a group of noncoding RNAs with diverse functions that are becoming increasingly recognized. In this overview we will focus on the lncRNA content of EVs, and their potential application and utility as disease biomarkers, using hepatocellular cancer (HCC) as an example. HCC-derived EVs have been shown to have selective enrichment of lncRNA, and some of these such as TUC339, linc-RNA ROR and lincRNA-VLDLR have been implicated in tumor cell behavior [4]. Enrichment of some lncRNA within circulating EVs may offer protection from degradation. Although EV transfer of lncRNA may have pathophysiological relevance, the precise function of many lncRNA is not known. Moreover, the functional integrity and capability of lncRNA within EV remains to be established. However, these limitations in our understanding of the functional or pathophysiological role of EV lncRNA do not impact on their potential utility as disease biomarkers [5].

## The need for new biomarkers for liver cancer

HCC is the most common type of primary liver cancer in adults and is a leading cause of cancer-related deaths worldwide, resulting in close to 750 000 deaths worldwide every year [6, 7]. Within the United States, the prevalence of HCC has been increasing by 1.75% per year, which is a cause for concern [8]. The major established risk factors for HCC are cirrhosis, viral hepatitis B or C infection and nonalcoholic fatty liver disease. Conditions associated with chronic liver injury caused by diseases such as alcoholic liver disease, hemochromatosis, primary biliary cirrhosis and toxins such as aflatoxins are additional risk factors for HCC [6]. The majority of HCC are diagnosed at an advanced stage after clinical deterioration has occurred and when curative therapy is not possible. HCC is associated with poor survival. Although advanced cancers have a poor survival, with a 5-year survival rate of <10% from the time of diagnosis, the survival in patients with resectable early-stage disease is much higher with a 5-year survival rate approaching 70% [6]. Surgical resection has been shown to be most beneficial for tumors that are still small singular nodules <2 cm wide [9]. Early detection of HCC at a time when surgery for cure can be performed offers the best option to improve outcomes from HCC. Indeed, screening and surveillance for HCC in patients with HBV and cirrhosis have reduced mortality from HCC. Thus, surveillance strategies to identify early HCC in persons at risk is justified and warranted.

Diagnostic tools for the detection of early HCC include serum biomarkers, radiological imaging such as abdominal ultrasound (US), computed tomography (CT), and magnetic resonance imaging (MRI) and liver biopsy. Ultrasonography is frequently used for screening, but has poor sensitivity for detection of smaller tumors [9]. Widespread use of CT and MRI for screening is limited because of their cost or associated risks of radiation exposure [6]. The use of more specialized techniques such as angiography is not practical for screening and surveillance [10]. Liver biopsy is invasive, and the risk of needle tracking or extrahepatic spread makes it undesirable for use to diagnose smaller lesions that may be amenable to transplantation or surgical resection with curative intent.

Analysis of serum tumor markers is an attractive choice for screening for HCC. The most common marker used to detect HCC is alpha fetoprotein (AFP). Serum AFP levels are elevated in some, but not all persons with HCC, and AFP levels >400 ng/ml are highly correlated with HCC [6]. However, AFP lacks the sensitivity necessary for accurate diagnosis and often misses small early-stage tumors. Furthermore, serum AFP levels can be elevated in patients with ongoing regeneration associated with hepatic injury in the absence of malignancy [7]. Although several other markers e.g. serum des-gamma carboxyprothrombin have been evaluated for HCC, they have not proven to be of greater utility, and thus, existing serum biomarkers lack adequate sensitivity and specificity for early detection of HCC. [6]. Therefore, a clear need exists for more effective biomarkers.

## LncRNA in liver cancer

The analysis of tumor-associated RNA within EVs could provide an opportunity for identification of novel biomarker candidates. While much attention has been given to aberrantly expressed tumor-associated protein-coding mRNAs, genome sequencing studies have identified deregulated tumor-associated expression of several noncoding RNAs such as miRNAs, and lncRNAs. In contrast to the extensive studies on mRNAs and miRNAs, our understanding of the role of lncRNAs in disease remains limited [11]. LncRNAs are genetically encoded RNA molecules that lack protein coding potential and are defined by a size >200 bp [11]. The majority of lncRNAs are transcribed by RNA polymerase II, and then undergo polyadenylation and pre-RNA splicing [12]. LncRNAs have been reported to have many diverse functions involved in regulation of gene expression through epigenetic regulation, chromatin modification, transcription or posttranscriptional processing [13]. lncRNAs have emerged as important regulators of gene expression in many cancers [14]. The emerging relevance to cancer supports the utility of this class of RNA genes as cancer biomarkers. Alterations in expression of several lncRNAs have been recently reported in HCC. Selected examples are listed in Table 1 and are described herein [12, 14–16]. Recent studies have identified several lncRNA, such as TUC339, linc-VLDLR and lncRNA regulator of reprogramming (linc-RoR) that have been detected within HCC-derived EV [2, 4, 17].

**Table 1:** LncRNAs associated with hepatocellular carcinoma

LncRNA ID	Full name / gene description	Chromosomal location	Size (Kb)	Biological function	References
HULC	Highly up-regulated in liver cancer	Chr 6p24.3	0.5	Promotes HCC growth by inhibiting miR-372	[18–21]
HOTAIR	HOX antisense intergenic RNA	Chr 12q13.13	2.3	Promotes cell proliferation by silencing HOXD genes	[22–24]
MALAT1	Metastasis-associated lung adenocarcinoma transcript 1	Chr 11q13.1	8.7	Promotes cell invasion by regulating alternate splicing	[11, 25–27]
H19	Imprinted maternally expressed untranslated transcript	Chr 11p15.5	2.3	Promotes HCC growth through involvement with IGF2	[11, 14, 28, 29]
HEIH	High expression in HCC	Chr 5q35.3	1.7	Promotes HCC growth by interacting with EZH2 and repressing p15, p16, p21, and p57	[11, 30]
MEG3	Maternally expressed gene 3	Chr 14q32.3	1.8	Inhibits cell growth by interacting with PRC2 complex	[11, 16, 31, 32]
MVIH	Microvascular invasion in HCC			Promotes tumor growth by inhibiting PGK1 secretion	[11, 33]
TUC338	Transcribed ncRNA encoding uc. 338	Chr 12q13.13	0.59	Modulates cell growth by inhibiting p151NK4a and activating CDK	[13, 34]
Dreh	Down regulated expression by HBx	Chr 5		Inhibits cell growth and metastasis by repressing vimentin	[35]
LET	Low expression in tumor	Chr 15		Suppresses metastasis by destabilizing NF90	[15, 36]
HBx-LINE	Fusion of HBx with cellular long interspersed nuclear elements	Chr 8p11.21		Promotes HCC growth by activating Wnt/β-catenin signaling pathway	[15, 37]
Ftx	Encoded in the X-inactivation center, DIST regulator	Xq13.2		Promotes tumor growth by activating Wnt signaling pathway	[15, 38]
ATB	Activated by TGF-β	Chr 14		Promotes metastasis by binding IL-11. Induced by TGF-β	[39]
PVT1	Plasmacytoma variant translocation 1	8q24		Promotes cell proliferation by binding NOP2	[40]
PCNA-AS1	Antisense to proliferating cell nuclear antigen	20p12.3		Promotes HCC growth by regulating and stabilizing PCNA	[41]
LincRNA-RoR	lincRNA-regulator of reprogramming	Chr 18	22.8	Promotes tumor cell survival during hypoxic stress	[17]
TUC339	Transcribed ncRNA encoding uc. 339	Chr 12	1.2	Modulates tumor cell growth and adhesion	[4]

### Highly upregulated in liver cancer

This RNA gene was first identified as a lncRNA that was highly specifically upregulated in HCC [18]. Highly upregulated in liver cancer (HULC) inhibits miR-372 activity in an autoregulatory loop that reduces translational repression of its target gene, protein kinase A catalytic subunit beta (PRKACB), and in turn inducing the phosphorylation of camp response element-binding protein (CREB), a transcriptional factor that regulates HULC expression [19]. HULC is aberrantly expressed in HCC tissues and in the plasma of HBV-positive HCC patients [20]. Overexpression of HULC can be an indicator of lower survival rate [15]. HULC expression is upregulated in HBx-producing cell lines, and upregulation of HULC by HBx suppresses p18 activity and promotes HCC cell proliferation [21]. HULC has been detected with higher frequency in the plasma of HCC patients when compared with healthy controls, and with greater frequency in patients with higher Edmondson histological grades [20]. HULC thus has potential value as a biomarker of HCC [20].

### HOX antisense intergenic RNA

This lncRNA is significantly overexpressed in HCC tissues and liver cancer cell lines [16] and has also been implicated in many other cancers. HOX antisense intergenic RNA (HOTAIR) induces transcriptional silencing of homeobox D cluster (HOXD) genes by targeting the polycomb repressive complex 2 (PRC2) complex to the HOXD locus [22]. High HOTAIR expression was correlated with poor patient survival and tumor recurrence [23]. HOTAIR levels in HCC tissues are higher when compared with adjacent noncancerous tissue. HOTAIR may also have utility as a prognostic marker for predicting HCC recurrence following liver transplantation [24].

### Metastasis-associated lung adenocarcinoma transcript 1

Metastasis-associated lung adenocarcinoma transcript 1 (MALAT1) is a large lncRNA and is >8000 nucleotides in length [25]. This lncRNA has been found to be involved in tumor metastasis and is implicated in a wide range of cancers [14]. MALAT localizes to nuclear speckles and functions in regulation of alternate splicing by altering the levels of phosphorylated to dephosphorylated serine/arginine-rich (SR) proteins [26]. MALAT1 levels are highly upregulated in HCC, with a nearly 6-fold increase in HCC compared with normal liver tissue [27]. MALAT1 overexpression has also been linked to cancer metastasis and tumor recurrence in patients following liver transplantation [27]. Therapeutic treatments that target MALAT1 can decrease cancer cell viability following transplantation and could be of clinical value [11].

### H19 imprinted maternally expressed transcript

H19 is an imprinted gene that is normally expressed in fetal liver and placenta during embryonic development, but is repressed after birth in most tissues [11]. Overexpression of H19 in adults is indicative of tumor development and growth [14]. High expression of H19 has been shown to be associated with HBV-related HCC [28]. The chromosomal location of this lncRNA is adjacent to the insulin-like growth factor 2 (IGF2) gene, and the biallelic expression of H19 and IGF2 may play a causal role in the epigenetic mechanisms involved in tumorigenesis in HCC [29].

### LncRNA with high expression in HCC

High levels of LncRNA with high expression in HCC (lncRNA-HEIH) in HBV-related HCC are markedly associated with recurrence, and can function as an independent prognostic marker for survival [11]. LncRNA-HEIH is also associated with cancer recurrence, with high expression predicting a worse prognosis [12]. This lncRNA interacts with the enhancer of zeste homolog 2 (EZH2) and represses the activity of EZh2 target genes p15, p16, p21 and p57, which are all important cyclin-dependent protein kinase inhibitors [30].

### Maternally expressed gene 3

Maternally expressed gene 3 (MEG3) has been found to be highly expressed in normal tissue but is downregulated in a number of human tumors [11]. The expression of MEG3 is decreased in HCC tumor tissues compared with nonmalignant tissues [31]. Furthermore, enforced expression of MEG3 in HCC cells notably inhibited cell growth and increased apoptosis [31]. It has been suggested that the Delta-like 1 homolog (DLK1)-MEG3 locus is continually deregulated in HCC [32].

### LncRNA-microvascular invasion in HCC

Upregulation of microvascular invasion in HCC (MVIH) has been shown to be associated with increased microvascular invasion and metastasis in HCC tissues [33]. Furthermore, high levels of MVIH can serve as an independent predictor of poor recurrence-free survival following hepatectomy in HCC patients [33]. This lncRNA is located in the intron of the ribosomal protein S24 (RPS24) gene and encodes a protein that belongs to the S24E family of ribosomal proteins [11]. MVIH may activate tumor-inducing angiogenesis by inhibiting the secretion of phospho-glycerate kinase 1 (PGK1) [33].

### TUC338

This lncRNA encompasses a sequence that is highly conserved across species and markedly upregulated in HCC cells compared with nonmalignant cells [34]. TUC338 expression corresponds to disease stage suggesting a potential role during malignant transformation [34]. Targeting TUC338 can be an effective method of modulating HCC growth [13]. TUC338 functions in controlling the G1/S checkpoint of the cell cycle by inhibiting p161NK4a and activating cyclin D1 / cyclin-dependent kinases (CDK).

### LncRNA downregulated expression by HBx

Downregulated expression by HBx (Dreh) is significantly downregulated in HBV-related HCC, and is associated with poor disease prognosis [35]. This lncRNA targets the intermediate filament vimentin and alters normal cytoskeleton structure, and inhibits HCC growth and metastasis both *in vitro* and *in vivo* [35].

### Low expression in tumor

Low expression in tumor (LET) levels were found to be decreased in tumor samples of HCC patients when compared with normal tissue [36]. Downregulation of LET by histone deacetylation 3 mediated hypoxia-induced HCC metastasis [36]. LET binds to and destabilizes nuclear factor of activated T-cells 90 kDa (NF90), a double-stranded RNA-binding protein that is implicated in tumor growth and metastasis [15].

### LncRNA-Hbx-LINE

The expression of HBx-LINE, short for fusion of the HBV encoded X protein and human cellular long interspersed nuclear elements, is upregulated in HCC tissues and associated with poor patient prognosis, and thus could potentially serve as a predictor of patient survival [37]. The HBx-LINE1 sequence is transcribed by the HBx promotor, and involves a fusion of HBx with cellular long interspersed nuclear elements (LINEs) [37]. HBx-LINE functions in tumor growth by activating Wnt signaling and inducing nuclear localization of β-Catenin [15]. HBx-LINE levels are upregulated in HCC tissues, and associated with increased colony formation, cell migration and epithelial-mesenchymal transition of tumor cells [37].

### LncRNA-Ftx

This lncRNA is encoded within the X-inactivation center and has been implicated in HBV-associated HCC [38]. Ftx may function in HCC tumor growth by activating the Wnt signaling pathway [15]. The Ftx transcript encodes miR-545/374 a, both of which showed increased expression in tumor tissue compared with noncancerous tissue taken from patients with HBV-related HCC [15]. Gender differences in miR-545/374 a expression are recognized with male HCC patients having higher expression than females [15].

### lncRNA activated by TGF-β

lncRNA activated by TGF-β (lncRNA-ATB) is a mediator of the transforming growth factor (TGF-β) signaling pathway, and is highly expressed in HCC tissues [39]. LncRNA-ATB promotes the invasion-metastasis cascade by binding to Ilterleukin 11 (IL-11) and triggering STAT3 signaling [39]. ATB expression is upregulated in metastatic disease and associated with poor disease prognosis, which could make it a potential HCC biomarker as well as a candidate target for anti-metastatic therapies [39].

### Plasmacytoma variant translocation 1

Plasmacytoma variant translocation 1 (PVT1) was found to be highly upregulated in HCC tissues and was associated with poor clinical prognosis in patients [40]. PVT1 increases Nucleolar Protein 2 (NOP2) levels by enhancing the stability of NOP2 RNA-binding protein [40]. PVT1 may promote cell proliferation, cell cycling and the acquisition of stem cell-like properties in HCC cells.

### Proliferating cell nuclear antigen antisense lncRNA

The proliferating cell nuclear antigen antisense lncRNA (PCNA-AS1) transcript is located opposite to the PCNA gene, and may regulate PCNA activity via RNA hybridization [41]. This antisense lncRNA has been implicated in HCC growth and tumorigenesis [41]. PCNA-AS1 is significantly upregulated in HCC compared with paratumoral tissues both in vitro and in vivo. Patients with high levels of PCNA-AS1 were also more inclined to develop multiple tumors.

### lncRNA regulator of reprogramming

This lincRNA was first identified as an epigenetic regulator involved in pluripotency and lineage commitment, and is significantly upregulated in malignant hepatocytes as well as enriched within EVs derived from tumor cells [42]. Recent studies show that lincRNA-RoR expression is increased during tumor hypoxia [17].

### TUC339

This is an ultraconserved RNA that is highly enriched within EVs released from HCC-derived tumor cells and has been implicated in modulating tumor cell growth and adhesion [4].

The emerging data on lncRNA involved in HCC indicates the presence of several tumor-associated lncRNA, some of which have been functionally linked to processes involved in tumor growth. Given the large repertoire of transcribed lncRNA within the genome, many more lncRNA associated with HCC are likely to emerge. Once associations with the presence, behavior or outcomes of HCC are ascertained, these will represent attractive candidates for further evaluation as biomarkers of disease.

## Technologies for detection of circulating lncRNA biomarkers

**Figure 2:**
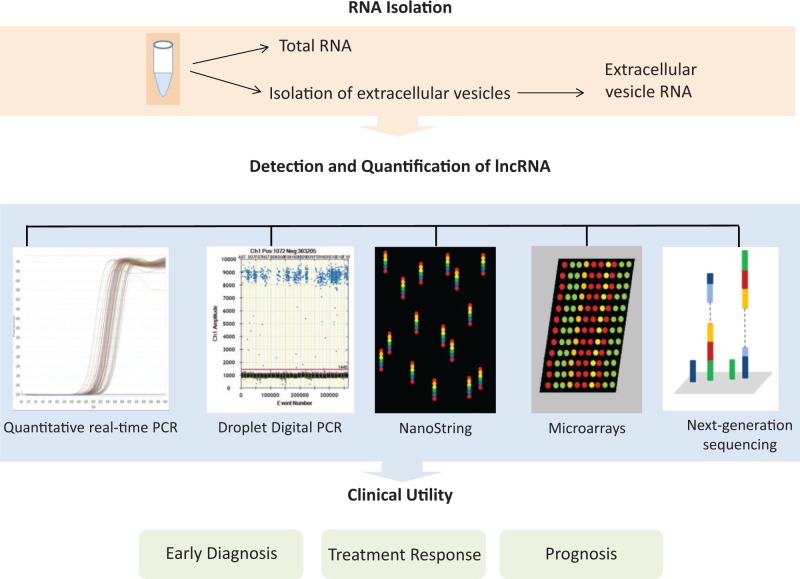
Overview of approach to analysis of circulating lncRNA as biomarkers of disease. A major determinant of success will be the availability of assays that have adequate sensitivity to detect small amounts of lncRNA in circulation, and knowledge of disease-specific lncRNA. (A colour version of this figure is available online at: http://bfg.oxfordjournals.org)

Accurate and sensitive detection of candidate lncRNA biomarkers in the circulation is a prerequisite for their use as biomarkers of disease or as markers to monitor disease progression, treatment effect or prognosis (Figure 2). Starting from blood or plasma, EV can be isolated using sequential ultracentrifugation or size-exclusion chromatography and EV RNA isolated [5]. Alternatively, total RNA can be isolated from blood samples. The latter will include both EV-associated RNA as well as free or protein-bound RNA. Several platforms are available for the detection of circulating RNA and include quantitative real-time polymerase chain reaction (qRT-PCR), droplet digital PCR (ddPCR), microarrays, nanostring and next-generation sequencing (NGS) [43] [Table 2]. However, the sensitivity of detection of the RNA transcripts of interest is the most important determinant. While tumor cells may release large amounts of EV, the absolute amount of a tumor-specific RNA in circulation will be extremely small.

**Table 2:** Platforms for detection of lncRNA

Technology	RNA quantity	Basic principle	Advantages	Disadvantages
qPCR	Nanograms	Amplifies a specific region of interest. Uses fluorescence signals to quantify PCR product.	Well-established and cost-effective technique. Less amount of starting material needed. Results are reproducible.	Requires reference genes and standard curves. Not effective at detecting small amounts of RNA.
dPCR	Nanograms	Partitions sample into multiple smaller reactions, performs amplification and detects ratio of positive to negative reactions.	Improved precision and accuracy. No need to depend on standard curves of endogenous controls. High sensitivity.	Expensive and lengthy procedure.
Microarrays	30 ng–5 µg	Probes are hybridized to fluorescent-labeled RNA. Probes are scanned and processed to detect RNA expression.	Able to simultaneously detect several genes. Can be customized.	Low sensitivity. Expression levels are lower for lncRNA.
NanoString nCounter Gene Expression Assay	Single cell	Probes are hybridized to target RNA. Machine digitally counts color-coded probe pairs to quantify gene expression.	Can detect multiple genes in a single reaction. High sensitivity and specificity.	Expensive technique.
NGS	1–5 µg	Template is created. Adaptors bind to gene of interest. Gene is amplified and sequenced.	High-throughput sequencing. Reduced cost and lessened sequencing time.	Shorter average read lengths. Data analysis is time-consuming and complex.

Microarrays are commonly used for RNA detection and are based on nucleic acid hybridization between target molecules and their complementary probes [44]. The signal intensities at their corresponding locations are used as a measure of relative RNA abundance [45]. Because microarrays consist of short length recognition sequences, each melting temperature (T_m_) is different, and this may negatively affect the specificity and sensitivity [46]. With the use of microarrays, the expression levels of lncRNA are also generally lower than protein coding genes [45]. An advantage of using microarrays is that it allows for the simultaneous detection of a large number of RNA genes, and arrays can be customized [46].

Quantitative PCR (qPCR) is a well-established tool for quantifying gene expression that relies on an increase in fluorescence signal that is proportional to the polymerase reaction product or amplicon [47]. Quantitative information is obtained from the cycle threshold (C_T_), which is defined as the PCR cycle at which the fluorescent signal of the reported dye crosses an arbitrary threshold [48]. The C_T_ value is inversely related to the amount of amplicon in the reaction; hence, a lower C_T_ value indicates greater gene expression [48]. Typically, C_T_ values for specific genes are referenced to well-known housekeeping genes such as GAPDH. However, this could be problematic because the housekeeping genes vary in expression between cell populations. The use of qPCR is limited because it requires an adequate standard curve to be generated for each sequence of interest [47]. Furthermore, this technique is not well-suited for detecting small amounts of RNA [43]. A limitation for the use of qRT-PCR for detection of EV RNA is the lack of information about housekeeping genes to enable quantitation across samples.

The NanoString nCounter Gene Expression Assay is a novel technology to measure RNA expression [46]. The assay is based on the direct detection of mRNA molecules of interest using target-specific, color-coded barcode probe pairs containing 35–50 base target-specific sequences. [49]. Digital analysis is then performed by quantitating barcodes [50]. This technology does not require amplification or reverse transcription, and because the counts are measured digitally, it is possible to detect small levels of RNA with high accuracy [46], and several hundred unique transcripts could be analyzed in a given reaction [50]. A comparison of the NanoString nCounter with microarrays and TaqMan PCR reported that the nCounter system is more sensitive than microarrays and similar in sensitivity to real-time PCR [49]. The sensitivity to detect EV RNA has not yet been determined.

Digital PCR (dPCR) offers an alternative and more direct approach for detecting gene expression; dPCR involves the partitioning of a sample into multiple separate reactions, such that some reactions contain no nucleic acid template and others contain one or more template copies [51]. The partitioned solutions undergo thermal cycling and end-point PCR. In ddPCR, droplets are generated and individual droplets analyzed using a fluorescence detector and used to determine the target concentration [43]. Unlike real-time PCR, dPCR allows for absolute quantification of a nucleic acid without the need for standard curves or endogenous controls. DPCR also offers improved precision and accuracy, thereby enabling smaller fold change differences to be detected [51]. DdPCR is ideal for the quantification of EV RNA samples, and is advantageous in studies where the target RNA concentration is low [47]. We have reported the use of dPCR for the detection of EV RNA.

NGS offers a powerful tool for detecting RNA molecules in biological samples. NGS initially requires the generation of a small RNA library in which the 5′ and 3′ RNA adaptors are ligated to either end of the noncoding RNA. The 3′ adaptors then bind to other small RNAs that carry the corresponding 3′ hydroxyl group. This is followed successively by reverse transcription and PCR amplification [46]. NGS is based on the concept of sequencing by synthesis; each DNA fragment to be sequenced is bound to an array, and DNA polymerase adds labeled nucleotides sequentially [52]. NGS is often referred to as ‘massively parallel sequencing' because it is capable of sequencing a large number of different DNA sequences in a single reaction [53]. A limitation of NGS is that it provides shorter average read lengths (30–400 bp) than conventional Sanger-based methods (400 bp–1 kb) [54], and these shorter reads may not align uniquely to the reference gene. The greatest advantage with NGS is the capability of producing a high volume of sequence data sets in the range of megabases to gigabases [54]. Reduced cost and shorter sequencing times are making NGS a more viable option for biomarker research [55]. However, the utility for detection of EV lncRNA remains to be established.

## Opportunities for lncRNA as disease biomarkers

An increasing number of lncRNA are being reported to be aberrantly expressed in human cancers. Characterization of these tumor-associated lncRNA offers the possibility of providing new insights into disease pathogenesis. lncRNA can be detected in EV released by tumor cells and can be detected in the circulation. However, the low expression of these in circulation and assays for clinical use will require highly sensitive detection technologies such as dPCR or next-generation sequencing. In addition to establishing tumor specificity, successful application of emerging knowledge of lncRNA will require the development of sensitive and reliable assays and a systematic demonstration of their clinical utility as biomarkers for disease diagnosis, prognosis, prediction of recurrence and therapeutic response. Such data are becoming available for several lncRNAs, for example HOTAIR and MALAT1 in predicting tumor recurrence following liver transplantation for HCC [16]. While the integrity and functional role of such lncRNAs and their presence within EV have yet to be established, the specificity of release from tumor cells within EV offers promise for their use as disease biomarkers.

Key Points

- Alterations in the expression of several lncRNAs such as HOTAIR and MALAT-1 have been reported in many cancers.
- Aberrant expression of lncRNA that is specific to certain tumors, such as HULC in hepatocellular cancer, support a role for these in disease pathogenesis.
- The differences in lncRNA present in tumor cells and in extracellular vesicles derived from these cells supports the existence of mechanisms that selectively enrich lncRNA within extracellular vesicles.
- New quantitative methods for RNA gene analysis such as digital PCR are enabling sensitive measurements of RNA within extracellular vesicles that will facilitate their adoption as cancer biomarkers.

## Funding

Supported by Grants DK 069370 and UH3-TR000884 from the National Institutes of Health.
